# Biofilm recruitment under nanofiltration conditions: the influence of resident biofilm structural parameters on planktonic cell invasion

**DOI:** 10.1111/1751-7915.12881

**Published:** 2017-12-01

**Authors:** Olivier Habimana, Eoin Casey

**Affiliations:** ^1^ School of Biological Sciences The University of Hong Kong Hong Kong China; ^2^ School of Chemical and Bioprocess Engineering University College Dublin (UCD) Belfield Dublin 4 Ireland

## Abstract

It is now generally accepted that biofouling is inevitable in pressure‐driven membrane processes for water purification. A large number of published articles describe the development of novel membranes in an effort to address biofouling in such systems. It is reasonable to assume that such membranes, even those with antimicrobial properties, when applied in industrial‐scale systems will experience some degree of biofouling. In such a scenario, an understanding of the fate of planktonic cells, such as those entering with the feed water, has important implications with respect to contact killing particularly for membranes with antimicrobial properties. This study thus sought to investigate the fate of planktonic cells in a model nanofiltration biofouling system. Here, the interaction between auto‐fluorescent *Pseudomonas putida* planktonic cells and 7‐day‐old *Pseudomonas fluorescens* resident biofilms was studied under permeate flux conditions in a nanofiltration cross flow system. We demonstrate that biofilm cell recruitment during nanofiltration is affected by distinctive biofilm structural parameters such as biofilm depth.

## Introduction

Membrane fouling remains as the leading obstacle to the efficient operation of pressure‐driven water filtration processes such as nanofiltration (NF) (Vrouwenvelder and van der Kooij, 2001; Gutman *et al*., 2012). The emergence of antifouling/antimicrobial (Mauter *et al*., 2011; Kroll *et al*., 2012; Liu *et al*., 2013; Seon *et al*., 2015) and non‐biocidal antifouling membrane technologies, such as zwitterionic coatings (Bernstein *et al*., 2011; Wibisono *et al*., 2015) and hydrogel modifications (Zhao *et al*., 2014), is expected to play a major role in the effective management on the biofouling problem. Although these antifouling technologies have typically been shown to retard, but not eliminate, biofilm growth, no studies, to our knowledge has gone as far as investigating the antifouling effectiveness of these membranes once covered by a biofouling layer, more specifically against incoming cells that have the potential to penetrate the biofouling layer. Moreover, unravelling the poorly elucidated internalization phenomena of free cells into established fouling layers under nanofiltration conditions could provide much‐needed insights into optimizing the antimicrobial functions and applicability of antifouling surface‐modified membranes. Therefore, it is within this context that this study aimed to investigate cell recruitment properties of 7‐day‐old biofilms under permeate conditions, with the goal of better identifying the level of planktonic cell internalization within biofilm models, differentiated by their structural properties. This was achieved by first creating a set of resident biofilms composed of *Pseudomonas fluorescens* cells grown for 7 days under nanofiltration conditions and fed with carbon source using two different loading rates, followed by interaction periods of relatively short timescales between introduced planktonic *Pseudomonas putida* cells and model resident biofilms under permeate conditions.

## Results and Discussion

### *Pseudomonas fluorescens* model resident biofilm recruitment using different carbon loading rates

The effect of carbon loading rates on *P. fluorescens* biofilm structural parameters was quantified following 7 days development under NF conditions (cf. Fig. S1 in Appendix), and results are presented in Table 1. Loading rates showed to have had an influence on biofilm structural properties. Compared to slow carbon loading rates (30 ml h^−1^), faster rates (50 ml h^−1^) were shown to result in distinctive structural biofilm outcome with higher biovolumes (*P *=* *0.029) and generated much thicker biofilms (*P *<* *0.0001). Other structural parameters such as porosity and biofilm roughness were shown not to have been affected by carbon loading rates (*P *>* *0.05).

**Table 1:** Structural and textural quantification of *Pseudomonas fluorescens* mono‐species biofilms following 7 days development under nanofiltration conditions at different carbon loading rates. The structural quantification of *P. fluorescens* biofilms was performed using ISA3D MATLAB‐based software, designed to perform automated quantification of biofilm structure through image analysis (Beyenal *et al*., 2004)

Mean	Biovolume (μm^3^)	Porosity^a^	Mean thickness (μm)	Biofilm roughness^a^	Textural entropy^a^	Textural energy^a^	Homogeneity^a^
Slow carbon loading rate	5.4 × 10^5^ ± 3.8 × 10^4^	0.8 ± 0.01	29.0 ± 2.3	0.3 ± 0.02	7.5 ± 0.3	0.03 ± 0.01	0.3 ± 0.02
Fast carbon loading rate	6.6 × 10^5^ ± 3.1 × 10^4^	0.8 ± 0.01	45.2 ± 3.2	0.3 ± 0.02	6.4 ± 0.5	0.04 ± 0.02	0.4 ± 0.05

a The selected structural and textural parameters are dimensionless, as described by Beyenal *et al*. (2004). Briefly, porosity is described as a ratio of void to total area, while biofilm roughness is used to depict the level of irregularities or break on a biofilm. For textural parameters, entropy, energy and homogeneity are a measure of randomness in pixel greyscale, directionality and spatial size of repeating pixel patterns, respectively.

Faster carbon loading rates may have provided an improved growth environment defined by the abundance of available carbon, allowing for faster optimized cell growth and consequently biofilm development, despite the harsh environment found on the membrane surface during NF processes (Habimana *et al*., 2014). While NF is used for the treatment of surface water, groundwater, treated wastewater and sea water (Hilal *et al*., 2004), which may differ in organic carbon variants (Nguyen *et al*., 2012), the importance of assimilable compounds during biofouling of membranes cannot be ignored. One earlier study by Hijnen *et al*. (2009) demonstrated that acetate concentrations as low as 1 μg C L^−1^ was sufficient for bacterial cells to adapt and thrive within NF processes (Hijnen *et al*., 2009). It was also shown that variations in acetate concentrations during filtration led to differentiations in fouling rates, where high acetate concentration led to higher fouling rates coupled with highest biomass cell counts (Hijnen *et al*., 2009), which confirms the differences in biofilm biovolume observed in this study.

Differences in mean biofilm thickness may reflect the degree of formation and accumulation of exopolymeric substances (EPS), in which a faster developing biofilm would yield more EPS. Nevertheless, biofilm textural analysis in this study provided clues of biomass properties following growth at different carbon rates. Textural entropy, which is a measure of randomness in the greyscale of images, was found to be lower for biofilms grown at faster loading rates (*P *=* *0.024), thereby implying less heterogeneous and more homogenous biofilms. No differences were, however, observed on the frequency of repeating cluster orientation and size patterns, presented as textural energy and homogeneity parameters (*P *>* *0.05), for biofilms grown under different carbon loading rates. This result suggests that mono‐species biofilms formed by *P. fluorescens* under NF conditions with different carbon loading rates may be depicted as globally similar, however, differing in overall biomass size.

### *Pseudomonas fluorescens* model resident biofilm recruitment of planktonic *Pseudomonas putida* cells under nanofiltration conditions

**Figure 1:**
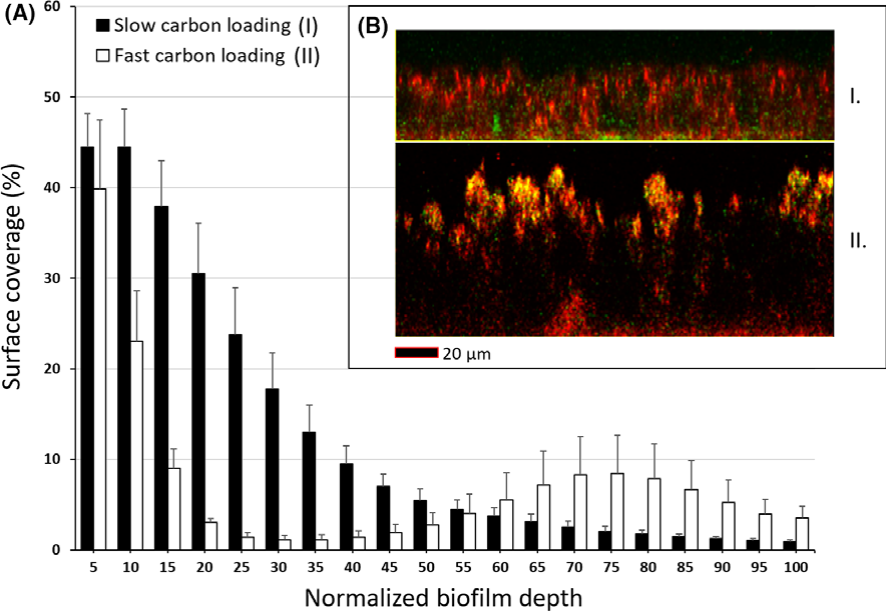
(A) Surface coverage (%) of internalized *Pseudomonas putida* cells within 7‐day‐old *Pseudomonas fluorescens* biofilms grown at different carbon loading rates. Surface area coverage versus biofilm thickness was performed using the PHLIP Matlab program. Error bars represent standard error of at least 15 independently acquired biofilm field of views taken during three independent experiments. (B) Maximum intensity images of 7‐day‐old *P. fluorescens* biofilms grown under slow (I.) and fast (II.) carbon loading rates under nanofiltration conditions. Red cells represent bacteria stained with Syto 61, and green and yellow cells represent *P. putida* cells expressing GFP and GFP together with Syto 61.

To assess biofilm reactivity to planktonic cells under relevant permeate flux conditions, a volume of 4 l feed solution spiked with *P. putida* cells was allowed to pass through the fouled system under nanofiltration conditions (cf. Fig. S1 in Appendix). Results indicate the *P. putida* cells were able to penetrate established biofilms under permeate conditions used in this study. Interestingly, when comparing the profile of internalized *P. putida* in established 7‐day‐old *P. fluorescens* biofilms in terms of surface coverage versus normalized biofilm depth (Fig. 1A), *P. putida* cells were predominantly embedded within the top and bottom biofilm layers grown under fast carbon loading rates. Under slower rates, the surface coverage was found to be much lower within the top regions of the biofilms, which increased as one approached the bottom of the *P. fluorescens* biofilm regions. *P. putida* embedded cells were qualitatively further substantiated by side view *xz*‐micrograph sections of *P. fluorescens* biofilms grown at different carbon loading rates (Fig. 1B). Moreover, biofilms grown under fast carbon rates were characterized with more zones with low fluorescence emissions indicating the high presence of EPS compared to biofilms grown under slower carbon loading rate conditions.

The differences observed in embedded *P. putida* cells could be linked to the level of resistance associated with the size of the fouling layer. In a recent and similar study, Heffernan *et al*. (2014) found that the presence of organic fouling layers on membranes during NF processes could act as a barrier to the penetration of planktonic cells to the surface of the membrane (Heffernan *et al*., 2014). Likewise, the difference in surface coverage with *P. fluorescens* biofilm thickness may possibly reflect some form of resistance based on biofilm structural disparities, such as biovolume and mean thickness biofilm parameters. In a similar type of study, under non‐permeate flux conditions, Habimana *et al*. (2009) showed that biofilm structural properties determined the degree of planktonic cell recruitment (Habimana *et al*., 2009). More specifically, they showed that porous‐type resident biofilms led to higher extent of planktonic cell internalization within the biofilm, while biofilms characterized with ample amounts EPS limited the internment of planktonic cells onto biofilms. Although permeate conditions used in this study most likely prompted contact interactions between planktonic cells and model biofilms, it was interesting enough to observe the limited penetrative behaviour of planktonic *P. putida* cells within biofilms grown in rich carbon environments, which may have resulted in generating biofilms having higher EPS fractions with distinctive properties. This shows that compositional and mechanical analyses of the EPS produced under different carbon loading rates would still be needed to provide a clearer understanding of the environmental factors linked to biofilm cell recruitment during NF processes.

## Conclusion

While it is generally well accepted that the biofouling phenomena is inevitable in pressure‐driven membrane processes for water purification, the internalization of cells under such processes remains poorly understood. This study set out to enquire about the role of an established biofilm layer in its interaction with incoming ‘fresh’ cells under nanofiltration processes. Our findings show that indeed, cell recruitment does occur in these systems, and that incoming cells can penetrate a resident biofilm; however, the degree of penetration of these cells is influenced by the structure of the existing biofilm. These observations will be important in developing robust experimental design methodologies for the assessment of antimicrobial/antifouling membranes. Future investigations should, therefore, focus on testing the effectiveness of antimicrobial nanofiltration membranes when obstructed with fouling layers constituted of organic matter or natural biofilms which would better represent industrial‐scale reality. The point of concern is the actual fate of these planktonic cells following internalization into fouling layers that may provide a protective and nutrient‐rich environment that could further perpetuate biofouling.

## Conflict of Interest

None declared.

## Supporting information


**Appendix S1.** Materials and Methods.
**Fig. S1.** Microbial Fouling Simulator cross flow system experimental rig.

## References

[mbt212881-bib-0001] Bernstein, R. , Belfer, S. , and Freger, V. (2011) Bacterial attachment to ro membranes surface‐modified by concentration‐polarization‐enhanced graft polymerization. Environ Sci Technol 45: 5973–5980. 21682251 10.1021/es1043694

[mbt212881-bib-0002] Beyenal, H. , Lewandowski, Z. , and Harkin, G. (2004) Quantifying biofilm structure: facts and fiction. Biofouling 20: 1–23. 15079889 10.1080/0892701042000191628

[mbt212881-bib-0003] Gutman, J. , Fox, S. , and Gilron, J. (2012) Interactions between biofilms and NF/RO flux and their implications for control‐A review of recent developments. J Membrane Sci 421: 1–7.

[mbt212881-bib-0004] Habimana, O. , Meyrand, M. , Meylheuc, T. , Kulakauskas, S. , and Briandet, R. (2009) Genetic features of resident biofilms determine attachment of *Listeria monocytogenes* . Appl Environ Microb 75: 7814–7821. 10.1128/AEM.01333-09 PMC2794110 19837841

[mbt212881-bib-0005] Habimana, O. , Semiao, A.J.C. , and Casey, E. (2014) The role of cell‐surface interactions in bacterial initial adhesion and consequent biofilm formation on nanofiltration/reverse osmosis membranes. J Membrane Sci 454: 82–96.

[mbt212881-bib-0006] Heffernan, R. , Habimana, O. , Semiao, A.J.C. , Cao, H. , Safari, A. , and Casey, E. (2014) A physical impact of organic fouling layers on bacterial adhesion during nanofiltration. Water Res 67: 118–128. 25265304 10.1016/j.watres.2014.09.012

[mbt212881-bib-0007] Hijnen, W.A.M. , Biraud, D. , Cornelissen, E.R. , and van der Kooij, D. (2009) Threshold concentration of easily assimilable organic carbon in feedwater for biofouling of spiral‐wound membranes. Environ Sci Technol 43: 4890–4895. 19673281 10.1021/es900037x

[mbt212881-bib-0008] Hilal, N. , Al‐Zoubi, H. , Darwish, N.A. , Mohammad, A.W. , and Abu Arabi, M. (2004) A comprehensive review of nanofiltration membranes: treatment, pretreatment, modelling, and atomic force microscopy. Desalination 170: 281–308.

[mbt212881-bib-0009] Kroll, S. , Brandes, C. , Wehling, J. , Treccani, L. , Grathwohl, G. , and Rezwan, K. (2012) Highly efficient enzyme‐functionalized porous zirconia microtubes for bacteria filtration. Environ Sci Technol 46: 8739–8747. 22827536 10.1021/es3006496

[mbt212881-bib-0010] Liu, Y. , Rosenfield, E. , Hu, M. , and Mi, B. (2013) Direct observation of bacterial deposition on and detachment from nanocomposite membranes embedded with silver nanoparticles. Water Res 47: 2949–2958. 23561495 10.1016/j.watres.2013.03.005

[mbt212881-bib-0011] Mauter, M.S. , Wang, Y. , Okemgbo, K.C. , Osuji, C.O. , Giannelis, E.P. , and Elimelech, M. (2011) Antifouling ultrafiltration membranes via post‐fabrication grafting of biocidal nanomaterials. ACS Appl Mater Interfaces 3: 2861–2868. 21736330 10.1021/am200522v

[mbt212881-bib-0012] Nguyen, T. , Roddick, F.A. , and Fan, L. (2012) Biofouling of water treatment membranes: a review of the underlying causes, monitoring techniques and control measures. Membranes (Basel) 2: 804–840. 24958430 10.3390/membranes2040804 PMC4021920

[mbt212881-bib-0013] Seon, L. , Lavalle, P. , Schaaf, P. , and Boulmedais, F. (2015) Polyelectrolyte multilayers: A versatile tool for preparing antimicrobial coatings. Langmuir 31: 12856–12872. 26513437 10.1021/acs.langmuir.5b02768

[mbt212881-bib-0014] Vrouwenvelder, J.S. , and van der Kooij, D. (2001) Diagnosis, prediction and prevention of biofouling of NF and RO membranes. Desalination 139: 65–71.

[mbt212881-bib-0015] Wibisono, Y. , Yandi, W. , Golabi, M. , Nugraha, R. , Cornelissen, E.R. , Kemperman, A.J.B. , *et al* (2015) Hydrogel‐coated feed spacers in two‐phase flow cleaning in spiral wound membrane elements: a novel platform for eco‐friendly biofouling mitigation. Water Res 71: 171–186. 25616114 10.1016/j.watres.2014.12.030

[mbt212881-bib-0016] Zhao, Y.F. , Zhu, L.P. , Yi, Z. , Zhu, B.K. , and Xu, Y.Y. (2014) Zwitterionic hydrogel thin films as antifouling surface layers of polyethersulfone ultrafiltration membranes anchored via reactive copolymer additive. J Membrane Sci 470: 148–158.

