# Supplementary material for: Biofilm recruitment under nanofiltration conditions: the influence of resident biofilm structural parameters on planktonic cell invasion
Source: Microb Biotechnol. 2017 Dec 1;11(1):264–7. doi: 10.1111/1751-7915.12881 (PMC5743815; doi:10.1111/1751-7915.12881)
Supplement: Supplementary file 1 — Appendix S1. Materials and Methods. Fig. S1. Microbial Fouling Simulator cross flow system experimental rig. [file MBT2-11-264-s001.docx]

**SUPPLEMENTARY INFORMATION**

**Materials and Methods**

Fluorescent mCherry-expressing *Pseudomonas fluorescens* PCL1701 and a GFP-expressing *Pseudomonas putida* PCL 1482 (Lagendijk, et al., 2010) were selected as model organisms for either generating model resident biofilms or to be used as planktonic cells during biofilm recruitment experiments respectively. *Pseudomonas fluorescens* biofilms were allowed to develop on NF90 thin film composite membranes (Dow Filmtec, USA) using a filtration setup designed for studying biofouling under NF conditions. Cell inoculum preparations and filtration system setup procedures for biofouling experiments are described in detail in similar recent studies (Habimana, et al., 2014, Semião, et al., 2014).

Briefly, *P. fluorescens* model resident biofilms were allowed to develop in a recirculating filtration system (Figure S1) in Raw water medium solution (Sodium bicarbonate (NaHCO_3_) 0.0042 gL^-1^, sodium chloride (NaCl) 0.0117 gL^-1^, potassium phosphate (KH_2_PO_4_) 0.0063 gL^-1^, magnesium sulphate (sold as heptahydrate, MgSO_4_**^.^**7H_2_O) 0.015 gL^-1^, ammonium chloride (NH_4_Cl) 0.005 gL^-1^, and calcium chloride (sold as dihydrate, CaCl_2_**^.^**2H_2_O) 0.0076 gL^-1^ , MilliQ water) for 7 days at a cross flow of 0.66 Lmin^-1^ per MFS (0.34 ms^-1^ cross flow velocity), a temperature of 20 ± 1 ºC, and a pressure inducing an initial permeate flux of 20 LMH (3 bar). These conditions were monitored and maintained for the duration of the experiments. During long biofilm experimental runs, fresh sterile Raw water solution was replaced every second or third day. Biofilm growth was promoted by supplying a carbon source into the feed tank throughout the 7-day NF process. A 0.006 mM sodium citrate (sold as tribasic dihydrate, Na_3_C_6_H_5_O_7_**^.^**2H_2_O; Sigma Aldrich, Ireland) solution was fed directly into the feed tank, via a Watson Marlow Sci-Q 400 peristaltic pump, at flowrates of either 30 mL hr^-1^ (12.96 µg C hr^-1^) or 50 mL hr^-1^ (21.6 µg C hr^-1^). The carbon source solution was maintained at pH 11 via drop-wise addition of 1 M NaOH to prevent bacterial growth within the carbon feed vessel. The addition of this alkaline solution did not affect the pH of the feed solution which remained constant throughout the duration of the experiments.

Following the 7-day biofilm growth period, a 200-mL suspension of *Pseudomonas putida* cells previously grown in raw water medium broth at 28 °C overnight was first centrifuged at 2772 RCF for 10 minutes using a Hettich Universal 320R centrifuge (Lennox, Ireland). Cell pellets were re-suspended in 10 mL PBS (corresponding to an inoculum of approximately 10^10^ cells mL^-1^).

The 10 mL *P. Putida* cell suspension was then used to inoculate the 10 L feed tank and the system immediately switched from recirculate mode to continuous mode, during which only 4 L was allowed to pass through the MFS operational system directly into a spent medium container at a cross flow velocity of 0.66 L min^-1^ per MFS (0.34 m s^-1^ cross flow velocity), a temperature of 20 ± 1 ºC, and a pressure inducing an initial permeate flux of 20 LMH (3 bar). This specific inoculation lasted about 3 minutes to complete. The filtration system was stopped and the removal of the membranes for microscopy preparation were as previously described by Semião *et al*., (2014) (Semião, et al., 2014). Briefly, the sample size for each membrane autopsy was about 1 cm^2^ of the middle section of the fouled membrane containing *P. fluorescens* biofilms and embedded *P. putida* cells, which were stained with 1 µL Syto 61 (5 mM, S-11343) (Molecular Probes) and incubated in the dark for 15 minutes prior to confocal microscopy. The importance of selecting two GFP and mCherry fluorescently-tagged model strains that could be simultaneously detected using confocal microscopy acquisitions was paramount in this experimental setup. It can be argued that there are many other possible micro-organisms equally or even more relevant than those used, however they are not generally available in a fluorescently-tagged form. To assess biofilms formed on the membrane, horizontal-plane images were acquired using an Olympus Fluoview FV 1000 Confocal microscope, previously set to automatically detect selected fluorophore emissions, within a spectral resolution of 1-2 nm. At least 5 to 6 random areas were acquired for each biofilm sample per experiment. The experiment itself was performed 3 times using independently grown bacterial cultures for each tested condition. The excitation wavelength used for detecting positively stained Syto 61 cells was 635 nm, and its fluorescence emission was recorded at 647 nm. Fluorescent GFP-expressing *Pseudomonas* cells were excited at 405 nm, and their fluorescence emissions recorded at 461 nm. Images were collected through an Olympus UPL SAPO 60x/1.40 Oil objective with a z-step of 1 μm. The field of view area for every acquisition was of 44931 µm^2^. 3D projections were performed using FIJI image processing tool’s 3D viewer plugin. The structural quantification of *P. fluorescens* biofilms was performed using ISA3D MATLAB based software developed by Beyenal *et al.,* (2004) (Beyenal, et al., 2004). The structural quantification of *P. putida* cells embedded within *P. fluorescens* biofilms in the form of surface area coverage versus biofilm thickness was performed using the PHLIP MATLAB program developed by J. Xavier (http: //phlip.sourceforge.net/phlip-ml) (Mueller, et al., 2006). The statistical significance of differences of the effect of carbon loading rates on biofilm structural parameters following 7 days nanofiltration on NF 90 was assessed using One-way analysis of variance with MINITAB v15.1 (Minitab Inc., State College, PA, USA). All tests were performed at a 5% significance level.


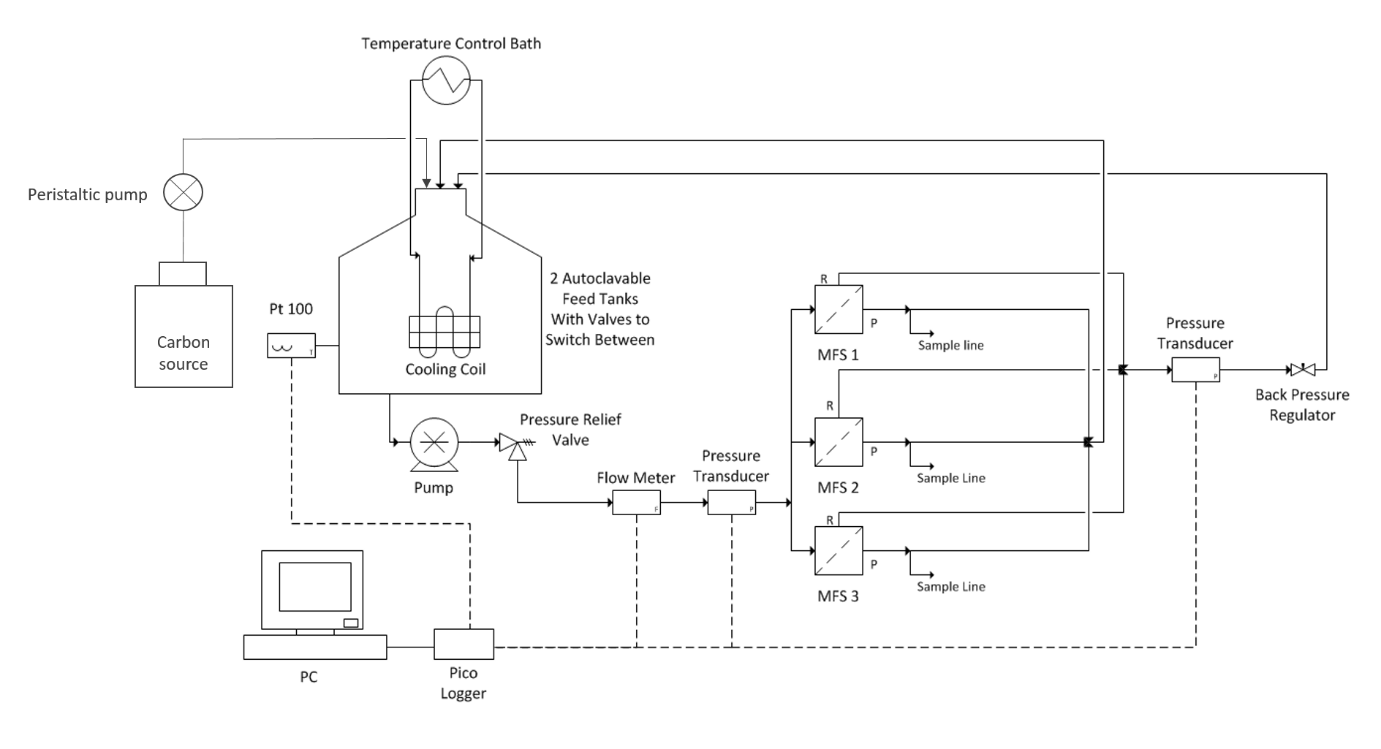


**Figure S1:** Microbial Fouling Simulator cross flow system experimental rig

**References**

1 Lagendijk, E.L., Validov, S., Lamers, G.E., De Weert, S., and Bloemberg, G.V. (2010) Genetic tools for tagging Gram-negative bacteria with mCherry for visualization in vitro and in natural habitats, biofilm and pathogenicity studies, *FEMS microbiology letters* **305**: 81-90.

2 Habimana, O., Semiao, A.J.C., and Casey, E. (2014) The role of cell-surface interactions in bacterial initial adhesion and consequent biofilm formation on nanofiltration/reverse osmosis membranes, *J Membrane Sci* **454**: 82-96.

3 Semião, A.J., Habimana, O., and Casey, E. (2014) Bacterial adhesion onto nanofiltration and reverse osmosis membranes: Effect of permeate flux, *water research* **63**: 296-305.

4 Beyenal, H., Lewandowski, Z., and Harkin, G. (2004) Quantifying biofilm structure: Facts and fiction, *Biofouling* **20**: 1-23.

5 Mueller, L.N., de Brouwer, J.F., Almeida, J.S., Stal, L.J., and Xavier, J.B. (2006) Analysis of a marine phototrophic biofilm by confocal laser scanning microscopy using the new image quantification software PHLIP, *BMC Ecol* **6**: 1.
